# Does the carer support needs assessment tool cover the established support needs of carers of patients with chronic obstructive pulmonary disease? A systematic literature search and narrative review

**DOI:** 10.1177/0269216320939243

**Published:** 2020-07-16

**Authors:** Kerry Micklewright, Morag Farquhar

**Affiliations:** School of Health Sciences, University of East Anglia, Norwich, UK

**Keywords:** Palliative care, caregivers, chronic obstructive pulmonary disease, systematic review

## Abstract

**Background:**

Informal carers play a key supportive role for patients with chronic obstructive pulmonary disease. However, caring can have a considerable impact on health and wellbeing. Carers may have unidentified support needs that could be a target for intervention. Literature on the support needs of informal carers has not been fully synthesised, and our knowledge of the comprehensiveness of the Carer Support Needs Assessment Tool for these individuals is limited.

**Aim:**

To explore whether the Carer Support Needs Assessment Tool covers the support needs of carers of patients with chronic obstructive pulmonary disease identified in published literature.

**Design:**

English language studies were identified against predetermined inclusion/exclusion criteria through database searching. Further studies were identified through searching reference lists and citations of included papers. Papers were critically appraised and data extracted and synthesised by two reviewers. Identified needs were mapped to Carer Support Needs Assessment Tool questions.

**Data sources:**

MEDLINE, CINAHL, EMBASE, CDSR, ASSIA, PsycINFO and Scopus databases (Jan 1997–Dec 2017).

**Results:**

Twenty-four studies were included. Results suggest that carers have support needs in a range of domains including physical, social, psychological and spiritual. Many of these needs are unmet. Particular areas of concern relate to prolonged social isolation, accessing services, emotional support and information needs. Findings also suggest amendment of the Carer Support Needs Assessment Tool may be required relating to difficulties within relationship management.

**Conclusion:**

Evidence suggests that carers of patients with chronic obstructive pulmonary disease would benefit from identification and response to their support needs by healthcare professionals but to enable this, the Carer Support Needs Assessment Tool requires an additional question. Future planned work will explore this with carers of patients with chronic obstructive pulmonary disease.

**What is already known about the topic?**

- Informal carers play a vital role in supporting patients with chronic obstructive pulmonary disease (COPD).
- COPD carers may have unidentified support needs that could be a target for intervention by clinicians.
- The Carer Support Needs Assessment Tool (CSNAT) has been developed as an evidence-based, person-centred approach to identify carer support needs, though as it was initially developed mainly with carers of patients with end-stage cancer, it is unclear if it encompasses all the potential support needs of COPD carers.

**What this paper adds?**

- Synthesis of knowledge relating to COPD carer support needs from published literature, including needs carers felt were met, needs carers felt were unmet and supportive inputs carers considered helpful.
- Mapping of these support needs to the CSNAT, which suggests that the addition of a question encompassing relationship management issues may be required to make it more comprehensive for COPD carers.

**Implications for practice, theory or policy**

- Many of the support needs of COPD carers are unmet; particular areas of concern relate to prolonged social isolation, accessing services, emotional support and information needs.
- COPD carers would benefit from identification, a comprehensive, person-centred assessment of their needs and appropriate response to these needs by clinicians.
- The CSNAT is a promising approach to identify COPD carer support needs, though it may require amendment to ensure it fully encompasses the potential needs of this group.

## Introduction

Informal carers are unpaid ‘lay people in a close supportive role who share in the illness experience of the patient and who undertake vital care work and emotion management’.^1^ They provide essential support to patients with progressive long-term conditions such as chronic obstructive pulmonary disease (COPD) relieving pressure on over-stretched health and social care services: one study reported that 77% of advanced COPD patients indicated they had a carer.^2^ Carers support patients with personal care, meal preparation, shopping, managing healthcare appointments, providing emotional support and administering medication, including the use and maintenance of nebulisers.^3–5^ Evidence also suggests that, historically, COPD patients have experienced significant inequalities in the provision of effective palliative care, potentially indicating the particular importance of informal care support for this group.^6–8^

However, informal carers are patients in their own right with health and support needs of their own.^9^ A breadth of research indicates that caring for patients with COPD can have considerable detrimental impacts on carers’ health and wellbeing. They report fatigue, anxiety, social isolation, depression, distress, financial difficulties and sleep disturbance.^5,10–13^ Meeting the support needs of these carers is essential if they are to manage their own health and wellbeing, while providing care throughout the trajectory of the patient’s illness. They may need support with both ‘direct’ needs, which refers to the support an individual may require for themselves, and ‘enabling’ needs, the support that carers may need to provide care.^14^ However, there are few evidence-based interventions to support COPD carers, who may particularly benefit from an effective and consistent process to identify and address their support needs.^13,15,16^

Policy indicates that the support provided to carers should be tailored to the needs of the individual.^17^ Identifying carers is the first step in this process, then a comprehensive, person-centred assessment should inform the provision of effective holistic support.^18^ One intervention used for this purpose in practice is the Carer Support Needs Assessment Tool (CSNAT) Intervention.^14^ The CSNAT Intervention comprises two components: (1) an evidence-based, 14 question tool that encompasses both direct and enabling needs which is integrated into (2) ‘The CSNAT Approach’, a five-stage person-centred process that facilitates a consistent and comprehensive assessment, response and review process. A strength of the CSNAT Intervention is that self-report is used to identify carers’ needs directly rather than clinicians inferring what is needed through indirect measures such as carer burden questionnaires. However, the tool itself was originally developed with carers of patients in hospice care settings towards the end of life, predominantly caring for those with cancer diagnoses, therefore whether the questions comprehensively cover the support needs of carers of patients with COPD has not been examined. The disease trajectory, symptoms, length and type of caring and the availability of patient and carer support can differ significantly between COPD and end-stage cancer.^19^ Investigation and synthesis of pre-existing evidence on COPD carer support needs may help to elucidate whether the CSNAT encompasses all of the needs of COPD carers and is suitable for use in practice with these carers in its current form. This review therefore aims to systematically identify carer support needs in COPD from the published literature and to use these findings to examine the comprehensiveness of the CSNAT for COPD carers through question mapping.

## Methods

### Review question

Does the CSNAT comprehensively cover the published support needs of carers of patients with COPD?

### Study design

A systematic search of peer-reviewed English language research literature published between 1997 and 2017 was conducted by two reviewers. The data extracted were synthesised and mapped to CSNAT questions, then narratively described. This approach was taken for reasons of pragmatism, based on the proposition that researchers should use the philosophical and/or methodological approach that works best for the particular research problem that is being investigated.^20^ This pragmatic approach was in keeping with the very applied nature of the review question. The review followed a registered protocol (Prospero database: CRD42018087082, accessible at https://www.crd.york.ac.uk/prospero/display_record.php?ID=CRD42018087082). The PRISMA checklist and flowchart were used to ensure clarity in reporting of activity at the various stages of the review.^21^

### Study identification

Medline, EMBASE, CINAHL, ASSIA, PsycINFO, Scopus and the Cochrane Database of Systematic Reviews (CDSR) were searched for relevant papers. Initial scoping searches of the literature indicated that carers may have support needs across a variety of domains (e.g. physical, psychological, spiritual and social). As such, a range of databases across multiple disciplines were included. Database searching was supplemented through forward and backward reference searching of studies identified as relevant.

Scoping searches of multiple grey literature databases including COPAC, OpenGrey, Ethos and ProQuest resulted in minimal relevant material, prompting the decision to exclude grey literature from the review.

The full inclusion criteria and search terms for this review are included as supplemental material (Appendices A and B). To be eligible, studies had to include adult participants (18+) who are/were informal carers of people diagnosed with COPD. Papers also had to include information about the support needs of these carers and/or ‘helpful inputs’ that carers considered helpful in reducing or resolving their needs. The review excluded editorials, opinion pieces, case studies and non-empirical studies.

A broad range of search terms were used due to variations in how informal carers are referred to by themselves and others: for example, spouses do not always identify with the term ‘carer’ as the support they provide is sometimes regarded by them as a natural extension of the marital role.^22^

### Data extraction

The first reviewer (K.M.) screened the titles and abstracts of potentially relevant papers against the inclusion criteria. If eligibility remained unclear, the full text was read; for any papers where uncertainty remained, the paper was discussed with the second reviewer to reach consensus. The second reviewer (M.F.) reviewed 10% of all identified papers to establish reproducibility of screening.

Data were extracted by the first reviewer. To refine this process, both reviewers independently extracted and interpreted data from three (12.5%) eligible papers initially. Findings were then compared and a consistent approach to the extraction and grouping of data was established.

For the purpose of this review, the meaning of ‘support needs’ was based on the Bradshaw’s concept of ‘expressed need’ given that the CSNAT is a tool used to identify the needs of a unique individual through self-report.^23^ Extraction of data on support needs was then based on the work of Ewing and Grande, creators of the CSNAT.^14^ Needs were thus identified and grouped in relation to

(1) Met needs: support needs that carers consider to be resolved, potentially through intervention from health or social services.
(2) Unmet needs: support needs that carers consider remain unresolved or unsupported.
(3) Helpful inputs: specific responses that carers considered useful in reducing or resolving their support needs.

The reviewers considered ‘needs’ and ‘responses to need’ in this way to separate discussion of support needs from discussion of potential interventions to meet these needs. This is because the appropriate response to a support need expressed by a carer may vary considerably depending on the individual. For example, two individuals may express a need for emotional support, but how this could be addressed depends on many factors, including the preferences of the individual and the local resources available.

### Quality assessment

Quality appraisal of eligible papers was carried out to identify potential areas of bias and consider the validity of findings reported within papers. Appraisal was completed by the first reviewer (K.M.). To determine accuracy of appraisal, the second reviewer (M.F.) additionally independently appraised a random sample (10%) of eligible papers. Minor differences were resolved through discussion.

Three quality appraisal tools were used, reflecting the varied methodology of included papers:

1. Qualitative: Items 1–9 of the Critical Appraisal Skills Programme (CASP) Checklist.^24^ Item 10 was excluded as all papers were judged to be of necessary value for inclusion in this review.
2. Quantitative: Appraisal Tool for Cross-Sectional Studies (AXIS).^25^
3. Narrative review: The reviewers modified the International Narrative Systematic Assessment (INSA) tool.^26^ Rather than using the point-scoring system devised by the creators of the INSA, items were phrased into ‘yes/no’ questions to maintain a consistent approach to quality appraisal.

### Data analysis

A licence granting permission to use the CSNAT within this review was obtained from its creators. Extracted ‘met needs’, ‘unmet needs’ and ‘helpful inputs’ were mapped onto the 14 CSNAT questions. In their study reporting the development of the CSNAT, Ewing and Grande explain how qualitative data from carers were initially grouped into discrete categories, which were further refined into CSNAT questions. The reviewers utilised these categories to ensure that mapping was congruent with the support needs Ewing and Grande intended to be encompassed by each question on the tool. This process was achieved by considering each identified need in turn and reviewing the CSNAT questions to identify whether, and where, the need could be mapped to the tool. Each of the individual CSNAT questions relates to a broad domain of need, therefore multiple support needs can map to one domain; individual needs can also sometimes map to more than one domain.

Extracted support needs that could not be mapped onto any of the tool’s questions indicated areas where amendment of the tool may be useful to ensure the CSNAT is comprehensive for COPD carers. Given the breadth of the individual CSNAT questions noted above, these unmapped support needs were then reviewed to establish whether they could be synthesised to represent one or more additional broad areas of support need (or new ‘domains of need’). This process was achieved primarily through discussion within the research team, followed by additional consultation with the CSNAT developers. This consultation included a revisiting and discussion of the developers’ original work in developing the tool plus work they were conducting (in parallel to the study reported here) to consider the utility of the tool with carers of patients with motor neurone disease (MND). Following this, a narrative description of the findings was produced. This design was congruent with paradigm of pragmatism in providing the best methodological approach to answer the very applied review question.^20^

## Results

### Study selection

**Figure 1.**
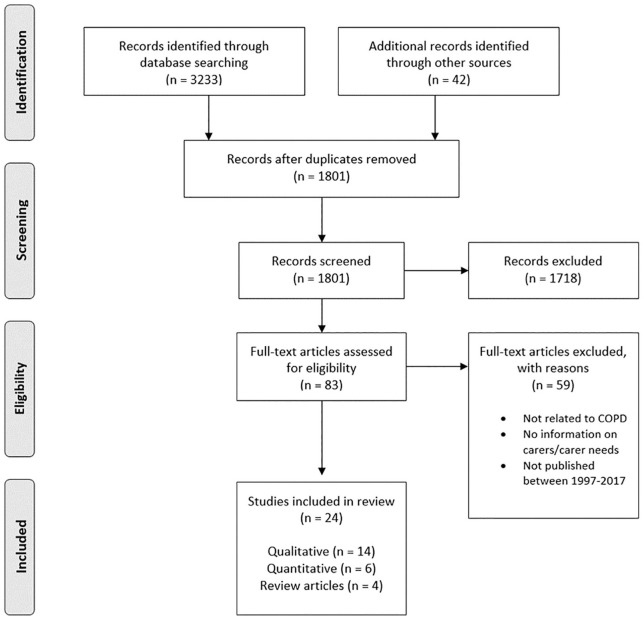
Preferred reporting items for systematic reviews and meta-analyses flowchart for article selection.

Searches yielded 24 eligible papers (see Figure 1); 14 were qualitative, six were quantitative and four were narrative reviews (see Appendix C – supplemental material). Details relating to the quality appraisal of these papers are also included in the study characteristics tables.

Included papers were completed in the United Kingdom,^16,18,27–29^ Iceland,^30^ Portugal,^13,31,32^ Denmark,^33^ Japan,^34^ the Netherlands,^35^ Sweden,^36–38^ Australia,^39–42^ Ireland,^5,43^ Canada^44,45^ and Norway.^46^ Participants from qualitative and quantitative studies were recruited from a range of sites, including outpatient clinics,^27,36^ inpatient units/hospitals,^33,46^ primary care,^31^ a civil registry,^29^ the community,^40,41,44^ pre-existing studies^45^ or more commonly from multiple settings.^28,30,32,34,37–39,43^ One study recruited carers from medical records, but the setting was unspecified.^5^ Another did not state any setting.^42^

All but one qualitative study used interviews to gather data;^5,18,27–32,36–39,43,44^ focus groups were utilised in the remaining paper.^33^ Quantitative studies were observational, with most being cross-sectional; ^34,40–42,46^ the one exception to this was Ross and Graydon,^45^ who employed case-control. Carers were more likely to be female, with 15 papers either recruiting more women than men or specifically targeting female participants.^27–30,32,33,37,39–46^ Only one study with mixed gender participants had a greater proportion of men than women,^36^ though two further papers were focused on male carers exclusively.^31,38^ Four included papers were review articles.^13,16,18,35^

### Quality appraisal

Several issues were revealed through quality appraisal that may impact upon the validity of findings. Multiple studies encountered difficulties with recruitment and carer engagement, including Hynes et al.^43^ and Bove et al.^33^ Researchers suggested these issues may have arisen from the disparity between how some carers self-identify and how they are labelled by others; researchers expressed concerns that the individuals they had attempted to recruit did not identify with the ‘carer’ label and hence chose not to engage with the studies. Further issues encountered within the studies related to carers struggling to engage due to difficulties in finding the time to participate or because of an unexpected deterioration of the patient’s health. Many studies recruited carers through patients or required the explicit consent of the patient to proceed.

### Overview of findings relating to identified carer needs

The majority of support needs reported were unmet. There were four key areas initially identified with which carers needed support: informational, emotional distress, social isolation and access to services. These are outlined before the mapping of COPD carer support needs to CSNAT items is presented.

#### Informational

Insufficient information provision was a consistent finding of the review. Carers appeared to struggle with understanding COPD as a medical condition; their lack of confidence in their knowledge of COPD was evident throughout. Although two studies suggested that carers had received sufficient disease-specific information to meet their needs,^39,42^ eleven papers indicated significant shortfalls in the information provided to carers.^5,13,18,28–30,37,38,43–45^ One paper described how the provision of adequate information about COPD only took place in the last few days of the patient’s life.^39^

Carers also demonstrated considerable unmet needs in relation to information about managing symptoms. Carers reported the need for further information about the patient’s medication,^31,33,42^ managing COPD exacerbations,^28,39^ managing the patient’s anxiety,^28^ managing oxygen and health aids,^43^ maintaining the quality of life for the patient-carer dyad^28^ and help to know when the patient required hospitalisation.^39,45^ Knowledge about, and signposting to, available services was also a major unmet need for carers.^5,16,18,28,29,35,41,42^

#### Emotional distress

Many of the carers expressed unmet needs in relation to emotional distress. Carers in ten of the included papers discussed a range of powerful emotions that they had not received support to manage, including helplessness, powerlessness, anxiety, fear, sadness, anger, frustration, guilt, resentment, shame, grief, loneliness and uncertainty.^13,18,29,33,35,38,42–45^ Carers felt abandoned,^29,33,44^ struggled to ask for help from others^29,30^ and reported that they had no one to talk to.^34,42,44,45^ In some cases, emotional needs stemmed from fearing for the patient. Carers worried about what would happen if they became too ill to provide care.^31,42^ They also feared the patient dying, being particularly fearful of death by asphyxia.^29,32,45^

#### Social isolation

Carers expressed difficulties relating to social isolation in half of the papers included in the review, with their caring responsibilities preventing them from feeling able to leave the house, have a social life, engage in leisure activities or have time for themselves.^13,18,29,30,32,33,35,36,38,42,44,45^ Leaving the patient at home could provoke guilt, fear and anxiety, inhibiting carers from pursuing opportunities for socialising outside the home. It is likely that difficulties accessing other sources of care for the patient contributes to this,^31,34,42^ and some carers reported a desire for a break from caring^30^ and support to reduce their exhaustion and fatigue.^18,29,36,44^

#### Access to services

Findings suggest that COPD carers encounter considerable barriers to accessing services. Carers appear to struggle with accessing financial assistance,^5,13,29,32,42–44^ alternative sources of care for the patient,^31,34,42^ support from social services,^13,46^ adequate specialist healthcare services,^31^ spiritual support^40,42^ and affordable, consistent home help.^18,34,37–39,42,43^ Studies also revealed significant difficulties in accessing equipment and housing adaptations.^5,18,28,29,39,44^ Interestingly, carers sometimes indicated that a patient struggling to access services also resulted in an unmet carer support need; for example, carers reported that a lack of patient access to emotional support and rehabilitation was an unmet carer need.^34,41^

### Mapping needs to CSNAT questions

The range of met needs, unmet needs and helpful inputs identified were then mapped to CSNAT questions. The outcome of this is reported below and summarised in Appendix D (supplemental material). Needs are grouped by the CSNAT questions they were mapped to during data analysis.

Most of the needs and helpful inputs extracted from papers were encompassed by the CSNAT, and no CSNAT questions appeared to be redundant for COPD carers. However, additional needs related to relationships were identified that could not be mapped to any existing CSNAT questions: the patient-carer relationship and the carer-clinician relationship. The majority of these relationship needs were unmet.

#### The patient-carer relationship

Carers in five studies reported needs that were related to changes or difficulties within the patient-carer relationship.^30,30,33,36,40^ Carers reported a loss of intimacy and understanding from the patient,^30,35,42^ tensions and constraints between them and the patient^32^ and a desire for health professionals to be receptive to discussing relationship problems.^38^

#### The carer–clinician relationship

Carers felt that clinicians did not always acknowledge carers and their role in supporting the patient.^28,36,43^ They reported contact with health professionals was inconsistent and that communication between carers and clinicians should be improved.^29,36,38,39^ Carers also reported a desire to be more included within decision-making regarding the patient’s care.^30,33,43^ Family meetings were suggested as one potential method to facilitate shared decision-making and understanding. Carers valued positive relationships with healthcare staff, placing particular emphasis on the importance of care that is well-coordinated between settings and led by empathetic staff.^35^

## Discussion

### Implications of unmet carer needs

This review primarily focused on the comprehensiveness of the CSNAT for COPD carers. However, several areas of shortfall in the support offered to COPD carers were also identified and are worthy of initial discussion. These areas included information provision, support for emotional distress, help to alleviate social isolation and assistance to access services.

Carers within the studies felt that the quality of information provided – and how it was transferred to them from healthcare professionals – must be improved. Many reported needing more information. The types of information needed varied widely. Examples included information related to the patient’s condition and its management as well as information to help establish an understanding of how services worked and interlinked. Among carers’ concerns was how a lack of information impacted upon on their understanding of COPD and hence how they cared for the patient. The finding that carers felt they lacked information is unsurprising. Research suggests that both carer and patient often receive inadequate information about COPD, its symptoms and treatments, sometimes to the extent that the patient is not even aware that they have been diagnosed.^47,48^ Ensuring that carers are included in information-sharing and signposting to further appropriate information resources by health professionals could be helpful in meeting their information needs. Professionals could also check that carers who have previously received information fully understand it. Research suggests that there is a notable disparity between the complexity of health information provided and the ability of patients and carers to comprehend it.^49^ Clinicians must be mindful that this may act as a barrier for carers to engage with health care resources, discussion with professionals and decisions relating to the patient’s care.

Interestingly, carers in one paper indicated that being offered information about COPD at multiple times as the disease progressed would have been helpful. This suggests that information tailored to the current disease stage – and hence to the carer’s current understanding – is the most beneficial. Carers also suggested that they would have liked to receive information both in the patient’s presence and independently, and that this may include information that the patient would prefer not to know at that time. For example, some of the patients within the papers did not want to receive information relating to how their disease will progress, end-of-life care or the dying process. However, this knowledge could help carers to plan for the future or prepare for the patient’s death and as such could be an important for carers. Some services have gone as far as to offer separate clinic appointments for carers and patients, acknowledging that the priorities and informational needs of these individuals may differ.^50^

The review revealed further areas where health professionals may be able to make a considerable difference for COPD carers. It appears that support to help carers engage socially and pursue interests outside the home would be of significant benefit to this group. Loneliness is a common issue among carers that is exacerbated by a range of factors, including financial difficulties and a lack of access to alternative sources of care for the patient.^51^ Given that unmet needs for financial assistance and input from formal care were also reported by carers in the review, it is possible that improving access to these may be helpful in reducing social isolation for COPD carers.

Health professionals could also play an important role in meeting the unmet need for emotional support revealed during the review. This support may involve promoting access to services that could help to reduce carer distress. Carers indicated that peer support or access to individuals to discuss their worries with – potentially including healthcare professionals – would be useful. Carers wanted staff to acknowledge carer distress and to demonstrate an understanding of the emotional impact caring can have on the wellbeing of carers.

There are other potential ways to address carer distress that healthcare professionals could consider. Given that access to services was a difficulty for carers within the review, ensuring that professionals are aware of appropriate signposting or local resources to help carers manage their mental health may be helpful, including wellbeing services, support lines or local support groups. Making sure carers are also aware of how to get help for the patient in an emergency – for example, establishing a contingency plan as to who would provide care should the carer become ill – also appears to be indicated given that this was noted as a specific source of distress and worry in two papers. It may be that managing emotions and worries could be linked to the aforementioned unmet need to reduce social isolation. Social isolation is associated with distress and poorer mental health outcomes, while social support – specifically emotional support – offers a protective effect against mental health conditions such as depression.^52,53^ By enabling carers to enrich their social lives, they may find they are able to engage in protective, meaningful activities and find others with whom they can share their worries. Healthcare professionals can take a proactive role in promoting services that enable carers to manage their emotions and to connect with others.

Effective palliative care also appears to have a potential role in reducing emotional distress for carers. Historically, the provision of palliative care for COPD patients and their carers has not been on par with the care provided for patients with other conditions.^6–8^ Aside from the previously mentioned information deficits regarding the future course of the illness and the dying process, carers also reported a variety of unmet emotional needs relating to end-of-life care, including the need for better access to bereavement support. Carers also reported fears and anxiety related to the patient’s impending death. This distress could potentially be relieved by support and discussion with appropriate palliative care professionals to help demystify end-of-life issues and prepare carers for what is to come.

A recurring finding was that clinicians may be able to improve the experience of carers by making them aware of, and facilitating access to, useful services. Carers reported a lack of signposting to services that could improve their quality of life or their ability to provide care. This included palliative care services, equipment services, experts who could provide assistance for financial and legal matters, affordable home help, peer support, services that could provide alternative sources of care for the patient and social care services. Health professionals could contribute to an improvement in signposting by ensuring that they are comprehensive in their assessment of the carer’s needs and are responsive in providing appropriate referrals and information for carers to access the services they require. Interestingly, studies also suggested that sometimes patients struggling to access services resulted in unmet support needs for carers; these included carers reporting a lack of patient access to rehabilitation and emotional support as an unmet carer support need. This may be related to the potential for these services to help reduce the burden on carers by maximising the function and independence of patients and through connecting patients to other individuals with whom they can share their worries. In terms of the benefits of rehabilitation to carers, research suggests that carers can derive relational and emotional benefits from services that help patients to increase their independence.^54^

In summary, a major finding of the review is the number of, and variety in, unmet support needs among COPD carers. It is likely that these individuals would benefit from identification of their needs in a timely and comprehensive way that leads to appropriate and tailored support from health and social care professionals. Utilisation of the CSNAT Intervention to identify support needs and develop an action plan to meet these needs may be one way of accomplishing this although, as we now discuss, the review also indicates that the CSNAT may need adaptation to more fully enable the support needs of COPD carers to be identified and addressed.

### Comprehensiveness of the CSNAT for COPD carers

Mapping the support needs of COPD carers to the CSNAT suggests that while none of the CSNAT questions are redundant, some adaptation of the tool may be required to ensure comprehensiveness for COPD, that is, that all potential COPD carer needs are encompassed.

The first category of support needs that did not appear to be covered by existing questions on the tool related to changes in the patient-carer relationship. It is worth considering that providing informal care can sometimes strengthen the patient-carer bond rather than leading to a deterioration in this relationship.^35^ However, for individuals experiencing relationship difficulties inclusion of this within the CSNAT may be beneficial, particularly as carers within the review suggested discussing relationship problems with health professionals may be a useful option. Interestingly, a similar finding was made by the CSNAT developers when investigating its potential utility with carers of patients with MND. While most MND carer support needs mapped onto CSNAT questions, the researchers also reported additional ‘relationship issues warranting further consideration’.^55^ It appears that MND and COPD carers may experience relationship difficulties that did not emerge in the original CSNAT study with predominantly end-stage cancer carers. There are a number of possible explanations for this. Differences in the respective trajectories and length of the caring role in these conditions may influence the patient-carer relationship over time in different ways, though no definitive conclusions regarding causation can be made without further investigation.

The discovery of support needs in relation to the carer-clinician relationship could also necessitate adaptation of the CSNAT. From the review, it appears carers sometimes experience difficulties within their interactions with health professionals which they struggle to resolve. Carers may benefit from being able to raise these concerns when completing the CSNAT to ensure that the support they are receiving is appropriate to meet their needs. As such, amendment of the CSNAT to encapsulate relationship difficulties may be appropriate to ensure it is comprehensive for COPD carers.

Given that both of these needs relate to relationships, it is proposed that the addition of a single appropriately worded question about relationships may address this.

### Limitations

This review utilised a systematic methodology and followed PRISMA guidelines^21^ to provide a comprehensive overview of COPD carer support needs. However, limitations remain, particularly in relation to sources of potentially relevant data that were excluded for pragmatic reasons. It is acknowledged that the inclusion of grey literature could have provided a more comprehensive evidence synthesis and might have reduced the impact of any publication bias on results.^56^ In addition, the exclusion of non-English papers and those published outside of the search period may mean that potentially relevant data were missed. Also, authors of papers included in the review were not contacted as field experts, which might have identified further literature not captured by the search.

Two further limitations relate to the nature of the empirical work in the included papers, which may indicate wider issues in carer research: (1) gender imbalance and (2) recruitment.

The majority of the participants within papers were female. This gender imbalance may reflect female carers outnumbering male carers in the general population.^57,58^ However, other factors related to study design may have affected recruitment. For example, male carers report that they have different needs to female carers and do not always identify with the ‘carer’ label, particularly if they are in employment.^59^ Hence, studies attempting to use the term ‘carer’ may have been more likely to alienate potential male participants. The inclusion of two papers with a sole focus on male carers increases the relevance of the review findings to this group, but the lower number of male participants in studies could reflect a gap in the literature that may be a useful area to address in future research on COPD carers.

In addition to this, several studies within the review struggled with recruiting carers. One study used a range of methods and sites to recruit but was unable to reach their recruitment goals.^43^ The study researchers considered whether this again may have been affected by a disparity between how carers self-identify and how they are labelled by others – specifically, whether individuals see themselves as ‘carers’. Many studies also recruited carers through patients or required the explicit consent of the patient to proceed. While pragmatic and understandable, this places patients in a ‘gatekeeping’ role which influence which carers were able to consider and participate in studies.^60^ For example, data might not be collected from carers where the patient-carer relationship is poor. Farquhar et al.^28^ acknowledge this limitation, though they also state that they felt the discovery of patient-carer tension in interviews was an encouraging sign that their recruitment approach had not compromised their findings. An additional difficulty encountered was the issue of carers having to balance study participation with their caring responsibilities, with some carers dropping out partway through the process as the patient became ill.^33,61,62^

### What this review adds

Four pre-existing review papers were identified and included within this review. Two of these, however, were narrative reviews which do not state a methodology or use systematic searching,^18,35^ The third investigated the impacts of caring and existing interventions rather than support needs,^13^ and the fourth had a search period of 1990–2007. This current review therefore adds a decade of published findings providing a more contemporary picture of support needs of COPD carers.^16^

This review forms one component of a study to explore aspects of validity of the CSNAT in COPD given its increasing use in this carer group. Follow-on empirical work with COPD carers will investigate the face and content validity of a revised CSNAT (revised to include a question about relationships) and explore their views on mechanisms for delivery of the CSNAT intervention in clinical practice to carers of patients with COPD.

## Conclusion

Individuals who support people with COPD have a range of support needs, many of which appear to be unmet. Health professionals could play an important role in enabling these needs to be addressed. However, needs must first be identified, and this could be accomplished through comprehensive person-centred assessment. The CSNAT may be a suitable tool to assess carer support needs; however, the current version of the tool would benefit from the addition of a question relating to relationships in order to be fully comprehensive of the needs of COPD carers.

## Supplemental Material

Appendix_A_Search_terms_121219 – Supplemental material for Does the carer support needs assessment tool cover the established support needs of carers of patients with chronic obstructive pulmonary disease? A systematic literature search and narrative review Click here for additional data file. Supplemental material, Appendix_A_Search_terms_121219 for Does the carer support needs assessment tool cover the established support needs of carers of patients with chronic obstructive pulmonary disease? A systematic literature search and narrative review by Kerry Micklewright and Morag Farquhar in Palliative Medicine

Appendix_B_Inclusion_criteria_121219 – Supplemental material for Does the carer support needs assessment tool cover the established support needs of carers of patients with chronic obstructive pulmonary disease? A systematic literature search and narrative review Click here for additional data file. Supplemental material, Appendix_B_Inclusion_criteria_121219 for Does the carer support needs assessment tool cover the established support needs of carers of patients with chronic obstructive pulmonary disease? A systematic literature search and narrative review by Kerry Micklewright and Morag Farquhar in Palliative Medicine

Appendix_C_Study_characteristics_table_020120 – Supplemental material for Does the carer support needs assessment tool cover the established support needs of carers of patients with chronic obstructive pulmonary disease? A systematic literature search and narrative review Click here for additional data file. Supplemental material, Appendix_C_Study_characteristics_table_020120 for Does the carer support needs assessment tool cover the established support needs of carers of patients with chronic obstructive pulmonary disease? A systematic literature search and narrative review by Kerry Micklewright and Morag Farquhar in Palliative Medicine

Appendix_D_CSNAT_mapping_table_230120 – Supplemental material for Does the carer support needs assessment tool cover the established support needs of carers of patients with chronic obstructive pulmonary disease? A systematic literature search and narrative review Click here for additional data file. Supplemental material, Appendix_D_CSNAT_mapping_table_230120 for Does the carer support needs assessment tool cover the established support needs of carers of patients with chronic obstructive pulmonary disease? A systematic literature search and narrative review by Kerry Micklewright and Morag Farquhar in Palliative Medicine
