# Supplementary material for: Does the carer support needs assessment tool cover the established support needs of carers of patients with chronic obstructive pulmonary disease? A systematic literature search and narrative review
Source: Palliat Med. 2020 Jul 16;34(10):1305–15. doi: 10.1177/0269216320939243 (PMC7543023; doi:10.1177/0269216320939243)
Supplement: Appendix_A_Search_terms_121219 – Supplemental material for Does the carer support needs assessment tool cover the established support needs of carers of patients with chronic obstructive pulmonary disease? A systematic literature search and narrative review [file Appendix_A_Search_terms_121219.docx]

| **Search terms** | | | | | | |
| --- | --- | --- | --- | --- | --- | --- |
| Disease  *‘OR’ between terms* |  | Interest |  | Population  *‘OR’ between terms* |  | Age group |
| (IN TITLE OR ABSTRACT)  COPD  Emphysema  Chronic Obstructive Pulmonary Disease | **AND** | (IN TITLE OR ABSTRACT)  Need* | **AND** | (IN TITLE OR ABSTRACT)  Carer* (allows carers)  Caregiver* (allows caregivers)  Supporter* (allows supporters)  Informal  Famil* (allows family, families, familial)  Friend* (allows friends)  Relative* (allows relatives)  Lay* (allows layman, laymen)  Spouse* (allows spouses, spousal)  Partner*  (allows partners) | **AND** | Adult |
| Expanders: similar terms * | | | | | | |
| Limiters: 1997-2017, English language, peer-reviewed, adult carers for those with COPD | | | | | | |

Appendix A: Table of search terms used in database searches.
