# Supplementary material for: Does the carer support needs assessment tool cover the established support needs of carers of patients with chronic obstructive pulmonary disease? A systematic literature search and narrative review
Source: Palliat Med. 2020 Jul 16;34(10):1305–15. doi: 10.1177/0269216320939243 (PMC7543023; doi:10.1177/0269216320939243)
Supplement: Appendix_B_Inclusion_criteria_121219 – Supplemental material for Does the carer support needs assessment tool cover the established support needs of carers of patients with chronic obstructive pulmonary disease? A systematic literature search and narrative review [file Appendix_B_Inclusion_criteria_121219.docx]

| **Inclusion criteria** | **Justification** |
| --- | --- |
| Participants who are/were informal carers of people diagnosed with COPD | All studies included need information regarding the experiences of this group to meet the aims of the review. Inclusion of former/bereaved carers may offer unique insights into the retrospective opinions of carers and how needs may change across the trajectory of the illness. |
| Papers in English only | Papers are in English only due to constraints in relation to time and finances. |
| Studies from between 1997-2017 | This review aims to provide insights into the contemporary situation for informal carers. |
| Peer-reviewed journal | Minimal relevant information was retrieved from grey literature databases. |
| Empirical research or review paper (qualitative, quantitative and mixed method reviews or studies) | This review aims to include as broad a range of relevant information as possible. |
| Paper includes information about the needs of informal carers for people with COPD, or inputs that they found or would find useful | Inclusion of this information is necessary to meet the aim of the review. ‘Input’ refers to a response to an identified need – for example, a carer being offered respite in response to a need to alleviate fatigue. |
| **Exclusion criteria** |  |
| Children/ adolescent participants (below 18 years) | The needs of child carers are likely to vary significantly from adult carers. As such, this group may benefit from an independent review that could inform a specialist support needs assessment tailored to this age group. |
| Paid/formal carers | This review focuses on informal/unpaid carers, whose experiences and support needs are likely to vary considerably from formal care staff. |
| Editorials, opinion pieces, case studies and non-empirical studies | This review excludes lower quality evidence to increase the reliability and strength of the findings. |

Appendix B: Inclusion and exclusion criteria.
