# Supplementary material for: Does the carer support needs assessment tool cover the established support needs of carers of patients with chronic obstructive pulmonary disease? A systematic literature search and narrative review
Source: Palliat Med. 2020 Jul 16;34(10):1305–15. doi: 10.1177/0269216320939243 (PMC7543023; doi:10.1177/0269216320939243)
Supplement: Appendix_C_Study_characteristics_table_020120 – Supplemental material for Does the carer support needs assessment tool cover the established support needs of carers of patients with chronic obstructive pulmonary disease? A systematic literature search and narrative review [file Appendix_C_Study_characteristics_table_020120.docx]

| Study | Country | Recruitment setting | Aim | Design | Data collection | Participant characteristics | Quality appraisal |
| --- | --- | --- | --- | --- | --- | --- | --- |
| **Qualitative** | | | | | | |  |
| Bergs, 2002^(1)^ | Iceland | Pulmonary unit in Reykjavík and an unspecified setting in Akureyri | To answer the question: ‘How do women caring for husbands with COPD experience their quality of life?’ | Qualitative: phenomenological | Interviews | Women who cared for husbands with COPD (n=6).  Age range: 47-69 years | 1. Yes 2. Yes 3. Yes 4. Can’t tell 5. Can’t tell 6. Can’t tell 7. Yes 8. Yes 9. Yes |
| Booth, Silvester and Todd, 2003^(2)^ | United Kingdom | Oncology and respiratory medicine clinics at a university teaching hospital | To investigate the experience of living with breathlessness in those suffering from advanced cancer or COPD and their carers. | Qualitative: thematic | Semi-structured interviews | Study included 10 COPD patients and their spouses (also 10 cancer patients and their carers).  M:F = 4:6  Age range: not reported | 1. Yes 2. Yes 3. Yes 4. Can’t tell 5. Yes, though data saturation not discussed 6. Can’t tell 7. Yes 8. Yes 9. Yes |
| Bove et al., 2016^(3)^ | Denmark | Department of pulmonary medicine within a hospital | To explore how spouses of patients with severe COPD experience their role as informal caregiver spouses. | Qualitative: thematic | Focus groups | Spouses of patients with severe COPD (n=22).  M:F = 9:13  Age range: 61-82 years  Average age: 69.4 years | 1. Yes 2. Yes 3. Yes 4. Yes 5. Yes, though data saturation not discussed 6. Can’t tell 7. Yes 8. Yes 9. Yes |
| Ek et al., 2011^(4)^ | Sweden | Pulmonary clinic | To illuminate couples’ experiences of living together when one partner has advanced COPD treated by means of long-term oxygen therapy. | Qualitative: phenomenological-hermeneutical | Interviews | 4 couples, half of whom were COPD carers (n=4).  M:F=3:1  Age range: 69-73  Average age: 70 | 1. Yes 2. Yes 3. Can’t tell 4. Yes 5. Yes (though data saturation not discussed) 6. Yes 7. Yes 8. Yes 9. Yes |
| Farquhar et al., 2017^(5)^ | United Kingdom | 10 primary care sites and 6 secondary oncology/respiratory sites | To identify the educational needs of carers of patients with breathlessness due to advanced disease in order to provide an evidence base for the intervention’s content. | Qualitative: thematic | Semi-structured interviews | 13 COPD patient-carer dyads (12 cancer dyads also included). Data about sex was combined across both groups (M:F=4:21).  Age range: 42-84 years  Average age: 68 years | 1. Yes 2. Yes 3. Can’t tell 4. Yes 5. Yes 6. Can’t tell 7. Yes 8. Yes 9. Yes |
| Figueiredo et al., 2015^(6)^ | Portugal | Two primary healthcare (1-3, 5, 7-18)centres | To explore the experience of husbands and sons providing care to a family member with moderate-to-severe COPD. | Qualitative: thematic | Semi-structured interviews | Carers were husbands (n=7) and sons (n=5) of COPD patients. Average ages:  Husbands: 70.9 ± 8.8 years  Sons: 43.4 ± 10.5 years | 1. Yes 2. Yes 3. Can’t tell 4. Yes 5. Yes, though data saturation not discussed 6. Can’t tell 7. Yes 8. Yes 9. Yes |
| Gabriel et al., 2014^(12)^ | Portugal | Primary care centre and a district hospital | To explore the perspectives of both patients and family members regarding the impact of COPD in their family life. | Qualitative: thematic | Semi-structured interview | Carers of those in the ‘chronic’ phase of COPD (n=20).  M:F=2:18  Age range: 38-81 years  Average age: 61.5±13 years | 1. Yes 2. Yes 3. Can’t tell 4. Yes 5. Yes, though data saturation and basis of questions used not discussed 6. Can’t tell 7. Yes 8. Yes 9. Yes |
| Hynes, Stokes and McCarron, 2012^(14)^ | Ireland | Primary care sites and community | To explore the experiences of informal caregivers providing care in the home to family members with chronic obstructive pulmonary disease. | Qualitative: hermeneutic phenomenological | Semi-structured interviews | Carers of patients with ‘advanced’ COPD (n=11).  M:F=2:9  Participants were between 20-79 years of age | 1. Yes 2. Yes 3. Yes 4. Yes 5. Yes, though basis of topic guide and data saturation not discussed 6. Can’t tell 7. Yes 8. Yes 9. Yes |
| Hasson et al., 2009^(13)^ | United Kingdom | Participants identified through a civil registry | To explore the palliative and end-of-life care needs and experiences of bereaved family members who cared for a person with advanced COPD in the home. | Qualitative: thematic | Semi-structured interviews | Carers for patients who died of COPD (n=9).  M:F=2:7  Age range: 25-65 years  Average age: 45-54 years | 1. Yes 2. Yes 3. Can’t tell 4. Yes 5. Yes 6. Can’t tell 7. Yes 8. Yes 9. Yes |
| Lindqvist et al., 2013^(15)^ | Sweden | 2 hospitals and a range of health care centres and patient associations | To describe conceptions of daily life in women living with a man suffering from COPD in different stages. | Qualitative: phenomenography | Semi-structured interviews | Women looking after a husband/cohabiting partner with COPD (n=21).  Age range: 53-84 years  Median age: 72 years | 1. Yes 2. Yes 3. Yes 4. Yes 5. Yes, though data saturation not discussed 6. Can’t tell 7. Yes 8. Yes 9. Yes |
| Lindqvist et al., 2013^(19)^ | Sweden | As above | To describe conceptions of daily life in men living with a woman suffering from COPD in different grades of the disease. | Qualitative: phenomenography | Semi-structured interviews | Men looking after a wife/cohabiting partner with COPD (n=19).  Age range: 55-85 years  Median age: 74 years | 1. Yes 2. Yes 3. Yes 4. Yes 5. Yes, though data saturation not discussed 6. Can’t tell 7. Yes 8. Yes 9. Yes |
| Philip et al., 2014^(16)^ | Australia | Tertiary outpatient clinics and a respiratory unit | To explore the experiences of current and bereaved informal carers of patients with severe COPD, with a focus of their information support needs and views of palliative care. | Qualitative:  thematic | Semi-structured interviews | A mix of current (n=9) and bereaved (n=10) carers.  M:F=9:10  Age range: 28-79 years (current), 52-83 years (bereaved)  Average age: 68 years (current), 73 years (bereaved) | 1. Yes 2. Yes 3. Yes 4. Yes 5. Yes 6. Can’t tell 7. Yes 8. Yes 9. Yes |
| Simpson et al., 2010^(18)^ | Canada | Community | To better understand the extent and nature of 'burden' experienced by informal caregivers in advanced COPD. | Qualitative: interpretive description | Semi-structured interviews | Carers for those with severe/moderate COPD being followed by a specific homecare team (n=14).  M:F=3:11  Age range: 46-89 years | 1. Yes 2. Yes 3. Yes 4. Can’t tell 5. Yes 6. Can’t tell 7. Yes 8. Yes 9. Yes |
| Spence et al., 2008^(20)^ | Ireland | Carers identified from medical records – setting not clear | To explore the specific care needs of informal caregivers of patients with advanced COPD. | Qualitative | Semi-structured interviews | Carers for patients with ‘advanced’ COPD (n=7).  F:M=1:6  Age range: 30-65 years (all but 1 stated to be 55-65) | 1. Yes 2. Yes 3. Can’t tell 4. Can’t tell 5. Yes 6. Can’t tell 7. Yes 8. Yes 9. Yes |
| **Quantitative** | | | | | | | |
| Currow et al., 2011^(7)^ | Australia | Community (participants recruited through an annual survey, the South Australian Health Omnibus) | To describe differences in caring and the support needed by caregivers of people with advanced ESLD compared to caregivers of people with other life-limiting diagnoses. | Quantitative: observational, cross-sectional study | Survey | Carers of patients with end-stage lung disease, among which were COPD carers (specific number unknown). From the total number of carers (n=145):  M:F = 55:90  Age: 31/145 were 65+ years old | 1. Yes 2. Yes 3. Yes 4. Yes 5. Yes 6. Yes 7. Yes 8. Yes 9. Yes 10. Yes 11. Yes 12. Yes 13. No 14. Not described, but data weighted using demographic data for whole population. 15. Yes 16. Yes 17. Yes 18. Yes 19. No 20. Yes |
| Currow et al., 2008^(8)^ | Australia | As above | To better understand the whole population of caregivers for people with ESLD leading to death. | Quantitative: observational, cross-sectional | Survey | Paper selected information about a certain subset of carers who cared for someone who died of COPD (n=104).  M:F= 40:64  Age range: 17-85 years  Mean age: 51.2 years | 1. Yes 2. Yes 3. Yes 4. Yes 5. Yes 6. Yes 7. Yes 8. Yes 9. Yes 10. Yes 11. Yes 12. Yes 13. No 14. Not described, but data weighted using demographic data for whole population. 15. Yes 16. Yes 17. Yes 18. Yes 19. No 20. Yes |
| Gautun, Werner and Lurås 2012^(9)^ | Norway | National hospital and a lung rehabilitation hospital | To answer who the informal caregivers of patients with COPD are, what kind of help they provide, and how they experience providing help to the patient. | Quantitative: observational, cross-sectional | Survey | Large sample of carers (n=545).  M:F=189:336  Average age: 59.2±13.2 years | 1. Yes 2. Yes 3. Yes 4. Yes 5. Yes 6. Yes 7. Unclear 8. Yes 9. The researchers produced a new questionnaire and validated it with a pilot group. 10. Yes 11. Yes 12. Yes 13. No 14. No 15. Yes 16. Yes 17. Yes 18. Yes 19. No 20. Yes |
| Ross and Graydon 1997^(17)^ | Canada | Participants recruited from pre-existing longitudinal study | To understand the impact on the wife of having a husband with COPD by determining the extent to which her mood was related to the stress of care giving, her health, her social support and the extent of the patient’s illness. | Quantitative: observational, case-control | Battery of research tools/scales | Women caring for their husbands with COPD (n=25). 25 ‘control’ wives also included.  Age range: 27-79 years  Average age: 65 years | 1. Yes 2. Yes 3. No 4. Yes 5. Yes 6. Yes 7. N/A 8. Yes 9. Yes 10. Yes 11. Yes 12. Yes 13. N/A 14. N/A 15. Yes 16. Yes 17. Yes 18. Yes 19. No 20. Yes |
| Takata et al., 2008^(10)^ | Japan | Medical centre and a hospital | To investigate the factors relating to depression among caregivers caring for outpatients with COPD. | Quantitative: observational, cross-sectional | Zarit Caregiver Burden tool, questions devised by authors | Carers for elderly COPD patients (n=45).  F:M=8:37  Average age: 68.0±11.0 years | 1. Yes 2. Yes 3. No 4. Yes 5. Yes 6. Selection process unclear 7. N/A 8. Yes 9. Yes 10. Yes 11. Yes 12. Yes 13. N/A 14. N/A 15. Yes 16. Yes 17. Yes 18. Yes 19. No 20. Is stated that patients and carers ‘agreed to participate’ – no further details given. |
| Woolfe, McMillan and Conway 2007^(11)^ | Australia | Setting not specified | To establish better local information about the needs of informal caregivers who provide care for patients with COPD at home. | Quantitative: observational, cross-sectional | Survey | Carers for COPD patients (n=63?)  M:F= 38.3%:61.7%  Age range: 34-83 years | 1. Yes 2. Yes 3. Yes 4. Yes 5. Yes 6. Yes, though convenience sampling used 7. Unclear 8. Yes 9. Yes 10. Yes, but not always clear why these were used 11. Yes 12. Yes 13. Unclear 14. Unclear 15. Yes 16. Yes 17. Yes 18. Yes 19. No 20. Yes |

Table 1: Study characteristics of included qualitative and quantitative studies.

| Study | Country | Aim | Design | Data collection | Participant characteristics | Quality appraisal |
| --- | --- | --- | --- | --- | --- | --- |
| **Reviews** | | | | | | |
| Caress et al., 2009^(21)^ | United Kingdom | To identify 1) The information and support needs of carers of family members with COPD; 2) appropriate interventions to support carers in their caregiving roles; 3) information on carers’ needs as reported in studies of patients living with COPD in the community. | Mixed methods narrative review | Database searching, reference list searches | Paper reported on a number of studies that included both male and female carers | 1. Yes 2. Yes 3. Yes 4. Yes 5. Yes 6. Yes 7. Unknown |
| Cruz, Marques and Figueiredo, 2017^(22)^ | Portugal | To provide an overview of the impacts of COPD on different levels of family carers’ lives and to search for interventions aimed at supporting them in their caregiving role. | Mixed methods narrative review | Database searching | As above | 1. Yes 2. Yes 3. Yes 4. Yes 5. Yes 6. Yes 7. None declared |
| Farquhar, 2017^(23)^ | United Kingdom | To outline considerations for the assessment process including what should be assessed, who should conduct assessment and what should happen as a result of assessment. | Qualitative narrative review | Not reported | As above | 1. Yes 2. Yes 3. No 4. No 5. Yes 6. Yes 7. None declared |
| Nakken et al., 2015^(24)^ | Netherlands | To review the current knowledge about informal caregiving in patients with COPD. | Narrative review | Not reported | As above | 1. Yes 2. Yes 3. No 4. No 5. Yes 6. Yes 7. None declared |

Appendix C: Table of study characteristics

1. Bergs D. ‘The Hidden Client’– women caring for husbands with COPD: their experience of quality of life. Journal of Clinical Nursing. 2002;11(5):613-21.

2. Booth S, Silvester S, Todd C. Breathlessness in cancer and chronic obstructive pulmonary disease: Using a qualitative approach to describe the experience of patients and carers. Palliative and Supportive Care. 2003;1(4):337-44.

3. Bove DG, Zakrisson A-B, Midtgaard J, Lomborg K, Overgaard D. Undefined and unpredictable responsibility: a focus group study of the experiences of informal caregiver spouses of patients with severe COPD. Journal of Clinical Nursing. 2016;25(3-4):483-93.

4. Ek K, Ternestedt B-M, Andershed B, Sahlberg-Blom E. Shifting Life Rhythms: Couples’ Stories about Living Together When One Spouse Has Advanced Chronic Obstructive Pulmonary Disease2011. 189-97 p.

5. Farquhar M, Penfold C, Benson J, Lovick R, Mahadeva R, Howson S, et al. Six key topics informal carers of patients with breathlessness in advanced disease want to learn about and why: MRC phase I study to inform an educational intervention. PLoS ONE. 2017;12(5).

6. Figueiredo D, Jácome C, Gabriel R, Marques A. Family care in chronic obstructive pulmonary disease: what happens when the carer is a man? Scandinavian Journal of Caring Sciences. 2016;30(4):721-30.

7. Currow DC, Farquhar M, Ward AM, Crawford GB, Abernethy AP. Caregivers' perceived adequacy of support in end-stage lung disease: results of a population survey. BMC Pulm Med. 2011;11:55-.

8. Currow DC, Ward A, Clark K, Burns CM, Abernethy AP. Caregivers for people with end-stage lung disease: characteristics and unmet needs in the whole population. International journal of chronic obstructive pulmonary disease. 2008;3(4):753-62.

9. Gautun H, Werner A, LurÅs H. Care challenges for informal caregivers of chronically ill lung patients: Results from a questionnaire survey. Scandinavian Journal of Public Health. 2011;40(1):18-24.

10. Takata S, Washio M, Moriwaki A, Tsuda T, Nakayama H, Iwanaga T, et al. Burden among caregivers of patients with chronic obstructive pulmonary disease with long-term oxygen therapy. International Medical Journal. 2008;15:53-7.

11. Woolfe P, McMillan M, Conway J. The needs of caregivers of people with COPD: A study. Australian Journal of Primary Health. 2007;13:28-35.

12. Gabriel R, Figueiredo D, Jácome C, Cruz J, Marques A. Day-to-day living with severe chronic obstructive pulmonary disease: Towards a family-based approach to the illness impacts. Psychology & Health. 2014;29(8):967-83.

13. Hasson F, Spence A, Waldron M, Kernohan G, McLaughlin D, Watson B, et al. Experiences and Needs of Bereaved Carers during Palliative and End-of-Life Care for People with Chronic Obstructive Pulmonary Disease. 2009;25(3):157-63.

14. Hynes G, Stokes A, McCarron M. Informal care-giving in advanced chronic obstructive pulmonary disease: lay knowledge and experience. Journal of Clinical Nursing. 2012;21(7‐8):1068-77.

15. Lindqvist G, Albin B, Heikkilä K, Hjelm K. Conceptions of daily life in women living with a man suffering from chronic obstructive pulmonary disease. 2012;14:40-51.

16. Philip J, Gold M, Brand C, Miller B, Douglass J, Sundararajan V. Facilitating Change and Adaptation: The Experiences of Current and Bereaved Carers of Patients with Severe Chronic Obstructive Pulmonary Disease. Journal of Palliative Medicine. 2014;17(4):421-7.

17. Ross E, Graydon JE. The Impact on the Wife of Caring for a Physically Ill Spouse. Journal of Women & Aging. 1997;9(4):23-35.

18. Simpson AC, Young J, Donahue M, Rocker G. A day at a time: caregiving on the edge in advanced COPD. International journal of chronic obstructive pulmonary disease. 2010;5:141-51.

19. Lindqvist G, Heikkilä K, Albin B, Hjelm K. Conceptions of daily life in men living with a woman suffering from chronic obstructive pulmonary disease. Primary Health Care Research &amp; Development. 2012;14(2):140-50.

20. Spence A, Hasson F, Waldron M, Kernohan G, McLaughlin D, Cochrane B, et al. Active carers: living with chronic obstructive pulmonary disease. International Journal of Palliative Nursing. 2008;14(8):368-72.

21. Caress A-L, Luker KA, Chalmers KI, Salmon MP. A review of the information and support needs of family carers of patients with chronic obstructive pulmonary disease. Journal of Clinical Nursing. 2009;18(4):479-91.

22. Cruz J, Marques A, Figueiredo D. Impacts of COPD on family carers and supportive interventions: a narrative review. Health & Social Care in the Community. 2017;25(1):11-25.

23. Farquhar M. Assessing carer needs in chronic obstructive pulmonary disease. Chronic Respiratory Disease. 2017;15(1):26-35.

24. Nakken N, Janssen DJA, van den Bogaart EHA, Wouters EFM, Franssen FME, Vercoulen JH, et al. Informal caregivers of patients with COPD: Home Sweet Home? European Respiratory Review. 2015;24(137):498.
