# Supplementary material for: Does the carer support needs assessment tool cover the established support needs of carers of patients with chronic obstructive pulmonary disease? A systematic literature search and narrative review
Source: Palliat Med. 2020 Jul 16;34(10):1305–15. doi: 10.1177/0269216320939243 (PMC7543023; doi:10.1177/0269216320939243)
Supplement: Appendix_D_CSNAT_mapping_table_230120 – Supplemental material for Does the carer support needs assessment tool cover the established support needs of carers of patients with chronic obstructive pulmonary disease? A systematic literature search and narrative review [file Appendix_D_CSNAT_mapping_table_230120.docx]

| CSNAT Question | Unmet needs: support needs that were not met for COPD carers | Met needs: support needs that were adequately met | Helpful input: a response or supportive input/intervention deemed helpful by carers |
| --- | --- | --- | --- |
| ***Support to enable the carer to care*** | | | |
| 1. Understanding your relative’s illness | The need for more disease-specific information about COPD that will help prepare carers for their caring role, including: information about the expected course of the disease, the potential for pneumonia and hospital admissions, the potential need for changes to the patient’s diet, the care the patient will require, long-term consequences of the diagnosis, skills training and an understanding of the term ‘COPD’(1-10)  Better knowledge and support from health professionals (e.g. full explanations of disease trajectory and management)(11)  A need for the dissemination of knowledge to carers to be consistent and well-planned(10)  Information specifically relating to breathlessness, including: what it is like to experience it, how it progresses and the variability of this symptom(3) | Information about the underlying reasons for symptoms and how to manage them (e.g. breathlessness)(12)  Information about therapies (e.g. breathing exercises)(12)  Information about ‘airways’(12)  The knowledge and confidence to deal with COPD through carer education *(Note: need was met only in the last few days of the patient’s life in this study)(13)* | Information about COPD and management techniques a) both together with the patient and separately, and b) on multiple occasions as the disease progressed(6)  Relevant information about COPD and treatment procedures(14)  COPD school for both patients and their partner, with a follow-up further in the future(7)  Pulmonary rehabilitation sessions(3, 4, 11) |
| 1. Managing your relative’s symptoms, including giving medicines | Support and knowledge on administering medication (and the effects of this medication)(12, 15, 16)  Knowing how to manage the patient’s anxiety and panic and how to respond helpfully to this(3)  Knowing how to manage exacerbations, infections and worsening breathlessness, including: when to start emergency medication, when to seek additional help, whether to increase steroids and having a response plan that reflects this(3, 13)  Help to know when to seek hospitalisation of the patient(8, 13)  Knowing how to manage oxygen and other health aids(5)  An educational intervention on breathlessness(3)  Training classes for caregiving(17)  Access to adequate specialist healthcare services(16)  Access to knowledge about how to maintain a reasonable quality of life for the patient-carer dyad and practical advice about helping the patient to be active(3)  Support for the carer that helps build confidence in managing the disease(3) | Effective and compassionate support from health professionals(4)  Health and social care support in sufficient quantity and quality, including GP visits *(Note: this was unmet for some carers in this study)(4)*  Feedback that carers are giving the patient appropriate care(12)  Finding ways to manage the patient’s decreased energy and nutritional needs(12)  Support to help the patient maintain independence and maximise their capabilities(12) | Help with the patient’s symptom control, medication and physical care(18)  Reorganising family routines to minimise the risk of exacerbating the patient’s symptoms(14)  Help and support from family, friends and professional networks(14)  Support from respiratory nurses(19) |
| 1. Providing personal care for your relative | Access to regular bathing services for the patient(17)  Support with physical care(20)  Confidence regarding the provision of personal care to the patient(16) | Help with the patient’s bathing(12) |  |
| 1. Knowing who to contact if you are concerned about your relative | A lack of knowledge about, and signposting to, available services(2-4, 10-12, 20-22)  Access to services for the patient, including rehabilitation and emotional support services(17, 20)  Access to services for the carer, including expert support (e.g. through guidance/counselling), support from social care (e.g. through an assigned social worker or a social welfare counsellor) and home help(7, 9, 10, 12)  Support that carers could contact if they felt they could no longer cope (e.g. a telephone hotline)(12)  A contact to answer questions and provide support, especially when the patient’s condition gets worse(7)  Better support from healthcare professionals, specifically someone who would ask how the carer is coping with all aspects of daily life(23) |  | Emotional support for the patient(18)  Support and access to health professionals, especially the primary care team(10)  Information about services available to the patient and carer(18) |
| 1. Equipment to care for your relative | Support to access to equipment to help care for the patient, including oxygen-related resources and equipment that assists with the patient’s mobility, showering and getting out of the house(2, 4, 9, 10, 13)  Access to housing adaptations (e.g. shower facilities)(4)  Information on what equipment is available and how to get it(3) | Equipment for the patient, including the instalment of a shower and a ‘special’ bed(7)  A need for car parking close to facilities(12) |  |
| 1. Talking with your relative about his or her illness | Help to talk to the patient about their condition(2) |  | Adequate communication between the patient and carer(22) |
| 1. Knowing what to expect in the future when caring for your relative | Support and information that enables planning and adaptation for the future, including: information about the future course of the illness and knowledge about the dying process(2, 10, 13, 20)  Support from, and knowledge about, palliative care services, including: discussion around prognosis, goals of care and support for both family and patient(13)  Help with managing anxiety about the future and being ready for ‘the end’(5, 11)  Involvement in making decisions concerning end-of-life issues and having carer views taken into account(4)  Support to help manage bereavement(22) | Knowledge and support from the palliative care team(11)  Support to manage bereavement (e.g. through counselling)(4) | Information regarding, and access to, bereavement support(4, 18)  Awareness of palliative care services(10)  Information about what to expect in the future and if the patient is likely to die within the year(18, 21) |
| ***Direct support for carers themselves*** | | | |
| 1. Having time for yourself in the day | Support to alleviate growing social isolation that encompasses: a loss of freedom, impaired social lives, not being able to leave the house, not being able to participate in leisure activities, not having time for themselves, not being able to spend time with other family members or away from the patient(1, 2, 4, 7-9, 11, 12, 14, 15, 22, 23)  Access to alternative sources of care for the patient (e.g. nursing home services, day care and other family members)(12, 16, 17)  Support to enable resting and a break from caring(1)  Help to alleviate feelings of fear, anxiety and guilt about leaving the patient(4, 9) | Having time for other family members(12) | Respite care to enable the carer to have time for themselves and to promote caring over the full disease trajectory(4, 8, 11)  Having informal support and respite through help from other family members(5)  Maintaining activities of personal interest and their own ‘space’(6) |
| 1. Your financial, legal or work issues | Financial assistance and help with issues due to loss of the patient’s income or expensive medication, advice on financial matters and assistance to pay for essential home renovations(4, 5, 9, 11, 12, 14)  Knowledge about how to access financial support, carer’s pension and possible benefit entitlements(10, 12)  Information about guardianship and power of attorney(12)  More flexibility and sympathy from employers to enable provision of care around working(16, 24) | Help with managing financial matters (e.g. paying bills or going to the bank)(12)  Grants for medication and medical bills(7) | Additional financial support(18)  Help to stay at work (an important form of respite from caring)(1) |
| 1. Dealing with your feelings and worries | Better emotional support to manage a range of emotions, including: helplessness, powerlessness, anxiety, fear, sadness, anger, frustration, guilt, resentment, worries, shame, grief, loneliness, uncertainty(2, 4, 5, 7-9, 11, 12, 15, 22)  Feeling able to ask for help from others, including family members(1, 4)  Help to reduce the impact of caring on mental health, including alleviation of depression, stress(6)  Help to manage emotions at particularly stressful times, including: at night, during a hospitalisation(19)  Managing fears relating to the patient’s impending death, including worries concerning death by asphyxia(4, 8, 14)  Accessible peer support or someone to talk to(8, 9, 12, 17)  Support to alleviate fears about not being able to provide care for the patient if the carer became ill(12, 16)  Help to alleviate feelings of abandonment by health professionals, including understanding from professionals and appropriate attention to the carer’s potential stress(4, 9, 15) | Help with mental health issues (e.g. GP support)(1)  Acknowledgement and emotional support from health professionals and the carer’s friends and family(12) | Emotional support for carers(18)  Participation in support groups and peer support(12, 21)  Being able to respect the patient’s wish to die at home(11)  Things that give security and peace of mind, including: alarm devices, encouraging test results(15)  Using positive reappraisal of the situation(14) |
| 1. Looking after your own health | Support to maintain the carer’s health and reduce exhaustion and fatigue(2, 4, 9, 23)  Support that enables resting and promotes carer self-care(9, 12)  Help to manage the carer’s declining mobility(22) |  | Advice on strategies to help maintain the carer’s health and wellbeing(7) |
| 1. Your beliefs or spiritual concerns | Spiritual support, including access to a religious leader to talk to and support from the carer’s religious group(12) | Personal prayer for strength and prayer from others(12) | Fulfilment of spiritual needs, including prayer(1)  Additional spiritual support(18) |
| 1. Practical help in the home | Additional, affordable help at home, for tasks including: help with housework, shopping, gardening and meal preparation/delivery(2, 4, 6-8, 10, 12, 13, 17)  A need for ‘outside help’ to be consistent(13)  Support to handle heavy oxygen equipment(23) | Additional practical support, specifically from formal carers(5) |  |
| 1. Getting a break from caring overnight | Help to manage difficulties sleeping, either due to supervising the patient or sounds of the oxygen machine(11) | A need to get enough sleep(12) |  |
| ***Support needs that may fall outside of the CSNAT*** | | | |
| 1. The patient-carer relationship | A need for more intimacy/closeness with the patient(1)  Understanding from the patient(12)  Help to manage changes in the patient-carer relationship, including: loss of intimacy, feelings of disgust due to coughing and impact of smoking on the relationship(22)  The option to discuss relationship problems with healthcare professionals(7)  The ability to manage tensions or constraints in a patient-carer couple relationship(14) |  |  |
| 1. The carer-clinician relationship | Improved, consistent, open communication with healthcare staff, preferably staff the carer knows and trusts(7, 13)  Support to foster inclusion in decision-making regarding the patient, including more family meetings with health personnel(1, 15)  Consistency with regards to contact with health professionals and the information they provide(4, 23)  Acknowledgment of the role of carers from healthcare professionals, including their knowledge and the support they provide the patient(3, 5, 23) |  | Watchfulness and participation in care and decision-making in the hospital context(5)  Care that is coordinated between care settings by empathetic and cooperative people(22) |

Appendix D: Table presenting the mapping of extracted carer support needs and helpful inputs to CSNAT items.

1. Bergs D. ‘The Hidden Client’– women caring for husbands with COPD: their experience of quality of life. Journal of Clinical Nursing. 2002;11(5):613-21.

2. Farquhar M. Assessing carer needs in chronic obstructive pulmonary disease. Chronic Respiratory Disease. 2017;15(1):26-35.

3. Farquhar M, Penfold C, Benson J, Lovick R, Mahadeva R, Howson S, et al. Six key topics informal carers of patients with breathlessness in advanced disease want to learn about and why: MRC phase I study to inform an educational intervention. PLoS ONE. 2017;12(5).

4. Hasson F, Spence A, Waldron M, Kernohan G, McLaughlin D, Watson B, et al. Experiences and Needs of Bereaved Carers during Palliative and End-of-Life Care for People with Chronic Obstructive Pulmonary Disease. 2009;25(3):157-63.

5. Hynes G, Stokes A, McCarron M. Informal care-giving in advanced chronic obstructive pulmonary disease: lay knowledge and experience. Journal of Clinical Nursing. 2012;21(7‐8):1068-77.

6. Lindqvist G, Albin B, Heikkilä K, Hjelm K. Conceptions of daily life in women living with a man suffering from chronic obstructive pulmonary disease. 2012;14:40-51.

7. Lindqvist G, Heikkilä K, Albin B, Hjelm K. Conceptions of daily life in men living with a woman suffering from chronic obstructive pulmonary disease. Primary Health Care Research &amp; Development. 2012;14(2):140-50.

8. Ross E, Graydon JE. The Impact on the Wife of Caring for a Physically Ill Spouse. Journal of Women & Aging. 1997;9(4):23-35.

9. Simpson AC, Young J, Donahue M, Rocker G. A day at a time: caregiving on the edge in advanced COPD. International journal of chronic obstructive pulmonary disease. 2010;5:141-51.

10. Spence A, Hasson F, Waldron M, Kernohan G, McLaughlin D, Cochrane B, et al. Active carers: living with chronic obstructive pulmonary disease. International Journal of Palliative Nursing. 2008;14(8):368-72.

11. Cruz J, Marques A, Figueiredo D. Impacts of COPD on family carers and supportive interventions: a narrative review. Health & Social Care in the Community. 2017;25(1):11-25.

12. Woolfe P, McMillan M, Conway J. The needs of caregivers of people with COPD: A study. Australian Journal of Primary Health. 2007;13:28-35.

13. Philip J, Gold M, Brand C, Miller B, Douglass J, Sundararajan V. Facilitating Change and Adaptation: The Experiences of Current and Bereaved Carers of Patients with Severe Chronic Obstructive Pulmonary Disease. Journal of Palliative Medicine. 2014;17(4):421-7.

14. Gabriel R, Figueiredo D, Jácome C, Cruz J, Marques A. Day-to-day living with severe chronic obstructive pulmonary disease: Towards a family-based approach to the illness impacts. Psychology & Health. 2014;29(8):967-83.

15. Bove DG, Zakrisson A-B, Midtgaard J, Lomborg K, Overgaard D. Undefined and unpredictable responsibility: a focus group study of the experiences of informal caregiver spouses of patients with severe COPD. Journal of Clinical Nursing. 2016;25(3-4):483-93.

16. Figueiredo D, Jácome C, Gabriel R, Marques A. Family care in chronic obstructive pulmonary disease: what happens when the carer is a man? Scandinavian Journal of Caring Sciences. 2016;30(4):721-30.

17. Takata S, Washio M, Moriwaki A, Tsuda T, Nakayama H, Iwanaga T, et al. Burden among caregivers of patients with chronic obstructive pulmonary disease with long-term oxygen therapy. International Medical Journal. 2008;15:53-7.

18. Currow DC, Farquhar M, Ward AM, Crawford GB, Abernethy AP. Caregivers' perceived adequacy of support in end-stage lung disease: results of a population survey. BMC Pulm Med. 2011;11:55-.

19. Booth S, Silvester S, Todd C. Breathlessness in cancer and chronic obstructive pulmonary disease: Using a qualitative approach to describe the experience of patients and carers. Palliative and Supportive Care. 2003;1(4):337-44.

20. Currow DC, Ward A, Clark K, Burns CM, Abernethy AP. Caregivers for people with end-stage lung disease: characteristics and unmet needs in the whole population. International journal of chronic obstructive pulmonary disease. 2008;3(4):753-62.

21. Caress A-L, Luker KA, Chalmers KI, Salmon MP. A review of the information and support needs of family carers of patients with chronic obstructive pulmonary disease. Journal of Clinical Nursing. 2009;18(4):479-91.

22. Nakken N, Janssen DJA, van den Bogaart EHA, Wouters EFM, Franssen FME, Vercoulen JH, et al. Informal caregivers of patients with COPD: Home Sweet Home? European Respiratory Review. 2015;24(137):498.

23. Ek K, Ternestedt B-M, Andershed B, Sahlberg-Blom E. Shifting Life Rhythms: Couples’ Stories about Living Together When One Spouse Has Advanced Chronic Obstructive Pulmonary Disease2011. 189-97 p.

24. Gautun H, Werner A, LurÅs H. Care challenges for informal caregivers of chronically ill lung patients: Results from a questionnaire survey. Scandinavian Journal of Public Health. 2011;40(1):18-24.
